# Multi-resource allocation and care sequence assignment in patient management: a stochastic programming approach

**DOI:** 10.1007/s10729-024-09675-6

**Published:** 2024-05-30

**Authors:** Xinyu Yao, Karmel S. Shehadeh, Rema Padman

**Affiliations:** 1 Carnegie Mellon University, Pittsburgh, PA USA; 2 Lehigh University, Bethlehem, PA USA

**Keywords:** Scheduling, Real-time location system data, Care pathway management, Uncertainty in activity duration, Stochastic programming, Operations research, Operations management

## Abstract

To mitigate outpatient care delivery inefficiencies induced by resource shortages and demand heterogeneity, this paper focuses on the problem of allocating and sequencing multiple medical resources so that patients scheduled for clinical care can experience efficient and coordinated care with minimum total waiting time. We leverage highly granular location data on people and medical resources collected via Real-Time Location System technologies to identify dominant patient care pathways. A novel two-stage Stochastic Mixed Integer Linear Programming model is proposed to determine the optimal patient sequence based on the available resources according to the care pathways that minimize patients’ expected total waiting time. The model incorporates the uncertainty in care activity duration via sample average approximation.We employ a Monte Carlo Optimization procedure to determine the appropriate sample size to obtain solutions that provide a good trade-off between approximation accuracy and computational time. Compared to the conventional deterministic model, our proposed model would significantly reduce waiting time for patients in the clinic by 60%, on average, with acceptable computational resource requirements and time complexity. In summary, this paper proposes a computationally efficient formulation for the multi-resource allocation and care sequence assignment optimization problem under uncertainty. It uses continuous assignment decision variables without timestamp and position indices, enabling the data-driven solution of problems with real-time allocation adjustment in a dynamic outpatient environment with complex clinical coordination constraints.

## Highlights

- We present a novel two-stage Stochastic Mixed-Integer Linear Programming model that addresses a complex clinical multi-resource allocation and sequencing problem.
- Our proposed model with continuous assignment decision variables without timestamp and position indices offers a solution to tackle this challenging problem effectively within reasonable computation time.
- The model addresses care delivery inefficiencies resulting from uncertain medical resource availability and variations in patient demand by minimizing the total patient waiting time.
- Our experimental results highlight the remarkable efficiency of our model, which successfully reduces outpatient waiting time by 60% by leveraging dominant patient pathways and their corresponding activity time distribution derived from in-hospital Real-time Location System tracking data. This demonstrates the potential impact of our approach in optimizing patient care management.

## Introduction

**Fig. 1:**
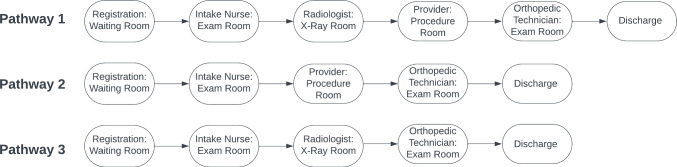
Illustration of possible patient pathways in an outpatient orthopedic clinic

In recent decades, and particularly highlighted during the COVID-19 pandemic, medical service congestion and care delivery inefficiencies induced by medical resource shortages and medical demand heterogeneity have posed an increasingly serious conundrum in healthcare environments [23, 38]. More than ever, well-designed resource management strategies are urgently needed to optimize diverse care pathways utilizing scarce resources for better medical care coordination [6]. A *care pathway* of a patient is a sequence of chronologically ordered care delivery activities that the patient experiences during the visit to a healthcare facility. This sequence often involves patient interaction with health professionals (e.g., medical staff, physician, nurse, technician, etc.) and utilizing various resources such as exam rooms and equipment. For example, Fig. 1 illustrates three possible patient care pathways in an outpatient orthopedic clinic from registration to discharge.

As pointed out by Villa et al. [53], efficient resource allocation and sequence assignment along patient pathways are vital to improving care coordination and patient experience. It provides a better understanding of the resources needed at each step of a patient’s journey through the system. By leveraging technology, data analytics, and evidence-based practices, healthcare providers can identify areas for improvement, track performance, and implement changes to optimize resource allocation and care delivery. Efficient resource allocation and sequence assignment ensure that the necessary resources are available when needed, resulting in timely patient care, reduced delays, cost savings, and ultimately, improved patient outcomes, thus enhancing the overall patient experience. In contrast, poor patient pathway scheduling with scarce resources could create bottlenecks in the healthcare system, causing cascading treatment delays and worsening patient outcomes. Unfortunately, resource-constrained care coordination is a well-known challenging optimization problem, especially when we consider heterogeneous medical resources with varying capacities needed by patients with uncertain service duration at each activity associated with their care process stages [49]. Therefore, it is crucial to understand different care pathway patterns to build a more efficient resource allocation and care sequencing optimization model.

The emergence of robust Real-Time Location System (RTLS) technologies and their recent deployments at scale by hospitals and health systems have opened up new opportunities to capture and collect highly granular time-stamped location data of the flow of patients, staff, and medical assets through the healthcare facilities [10, 24, 40]. Such granular data allows researchers and hospital administrators to identify sources of inefficiency and improve patient pathway modeling with better care coordination and asset management [16, 24]. In healthcare settings, RTLS technology has been employed for measuring patient density and resource utilization in hospitals [5], finding people and equipment to ensure safety [7], operational error control [56], refining the inputs for clinical simulation [39], and improving care coordination and safety policies [1, 24, 31, 40]. In addition to healthcare, RTLS has also been applied in other fields such as vehicle deployment planning [25] and warehouse management [14]. However, to the best of our knowledge, RTLSs have not been extensively employed to provide support, guidance, or modify operations while taking resource constraints into consideration [3].

**Fig. 2:**
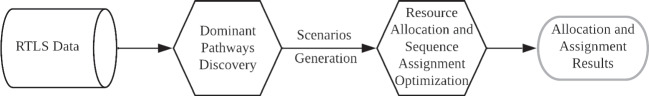
Schema: RTLS-data-enabled resource allocation and care sequence assignment framework

To address this difficult, unsolved problem, we propose an RTLS-data-enabled resource allocation and care sequence assignment framework for patient management and decision support. The framework’s objective is to minimize the expected total waiting time of all the patients within an acceptable time horizon. A schematic of our analysis framework is shown in Fig. 2. Specifically, the framework starts with leveraging RTLS data to identify dominant pathways of patient activities. These pathways, along with activity duration distribution information, serve as the core knowledge foundation for generating patient cohort scenarios, which subsequently become the input for the optimization module. The optimization module takes into account both capacitated heterogeneous resources and the uncertain service duration for patients at each activity stage. The specifics of the two modules are outlined below.

The Dominant Pathways Discovery is a data analytic and pattern-mining module to determine the major care pathways and their distribution among the patient population. This module plays an essential role in exploring the heterogeneity of patients’ care demands and translating care into medical resource demand for the subsequent optimization module. RTLS data enables the identification of patient navigation patterns from which dominant pathways are extracted by providing precise, real-time information on patients’ co-location sequences with specific types of medical staff and resources and their corresponding duration. Specifically, we leverage RTLS data to identify common care pathways and the service time distribution of each care activity in each pathway. The major challenges in this module include detecting and dealing with wrong RTLS records, carrying out data cleaning and prepossessing to transform raw RTLS logs to patient pathways, and fitting the care pattern distribution as well as service time distribution for each activity in different pathway patterns. Through these steps, we generate resource-demand scenarios to simulate the medical demand in the real world, which will be input parameters for the optimization module.

The Resource Allocation and Care Sequence Assignment Optimization module is constructed using an advanced stochastic programming model whose output is the suggested resource allocation and sequence assignments that minimize the total waiting time for patients in the clinical setting within an acceptable time horizon. In the optimization module, there are three major challenges associated with optimizing clinical care coordination. First, even for simplified scenarios that ignore the uncertainties in the system, the deterministic patient allocation and sequence assignment with multiple resources is complicated due to the heterogeneity of patient characteristics and limited resource capacities. Second, the randomness in service duration of care activities is significant. Ignoring such uncertainty may result in patient delays, poor utilization of limited resources, increased operating costs, and poor patient outcomes. Third, there is limited data to model such uncertainty due to the difficulties in gathering such detailed information and privacy issues [34]. As seen during the COVID-19 pandemic, health systems have struggled to provide quality care under limited resource availability and unpredictable patient length of stay in critical care units [35].

In summary, this paper focuses on resource allocation and sequence assignment modeling based on the learned dominant patient pathways. Specifically, the model will generate the solution of which and how many resources/staff will serve the patient at which visit stage and at what time. With our analytical model-driven decision support framework and optimization solution, hospitals and health systems will be able to obtain highly efficient resource assignment strategies for scheduling their patients in the clinical setting. Given the historical data on patient flows from using the RTLS technology, we assume that we can estimate patients’ future pathways as defined by the room, providers, and the resources they consume. Furthermore, we derive distributions for each activity duration along the patient pathways based on the real data. The proposed Resource Allocation and Sequence Assignment Module generates the optimal assignment solution in the dynamic environment by utilizing the activity sequence information along with the duration information for each stage for each pathway type.

The remainder of this paper is structured as follows. Section 2 provides a detailed literature review on optimization models for resource allocation and sequence assignment. Section 3.1 introduces the assumptions, parameters, and decision variables of the proposed models. Section 3.2 contains the complete formulation of the proposed model with a solution approach. The data, experiment settings and results are detailed in Section 4. Section 5 summarizes the research and concludes with a discussion of some limitations and future directions.

## Literature review

The clinical resource allocation and schedule sequencing problem has been an active research topic since the pioneering papers in the 1950s [55]. These early studies mostly assumed that patients are homogeneous concerning service time distribution and arrival pattern [11, 21]. However, consistent with precision medicine goals, researchers have more recently been developing resource allocation models that actively consider patient characteristics and needs to design more personalized solutions for healthcare delivery and management [41, 46]. Kong et al. [29] reported that utilizing the heterogeneity of patients is conducive to arrival order sequencing. Srinivas et al. [50] found that applying cluster scheduling based on grouped patients is appropriate for clinics to reduce appointment delays. Salzarulo et al. [43] further demonstrated how patient characteristics at the individual level could be used to predict patient service duration and support effective scheduling decisions. It is worthwhile to note that few studies have explored patient flow patterns using the highly granular data from RTLS for downstream scheduling or resource allocation.

The resource allocation and sequencing problem is a variant of the Job-Shop Scheduling Problem (JSP), which is well-known as one of the most challenging combinatorial optimization problems to solve using both exact and approximation methods [18]. Specifically, the NP-hard JSP considers the following task: there are *n* jobs to be processed through each of *m* machines exactly once with their predefined orders $\pi _i\ (i\in [n])$; for short, we call the event of job *i* processing on machine $j\ (j\in [n])$ as operations $o_{ij}$; the time duration required for $o_{ij}$ is known and denoted as $d_{ij}$; each machine can only process one job at a time, which means that the successor operations cannot start before the completion of their predecessors on each machine; an operation cannot be paused or interrupted once begun; the scheduling goal is to determine the optimal job sequence for each machine to minimize the total makespan or time to completion of the job [2].

To obtain an exact optimal solution for the JSP, mathematical programming models, particularly Mixed Integer Linear Programming (MILP) models, are the most common approach. There are three standard formulation categories for JSP, that is, the time-indexed formulation [8], the rank-based formulation [54], and the disjunctive formulation [36]. Ku et al. [30] showed that disjunctive models perform best for JSP with commercial Integer Programming software such as CPLEX or Gurobi regarding the Mean Relative Error performance over time. However, none of these models can be directly modified to solve the medical resource allocation and staff sequencing model in this study.

The general resource allocation problem can be deemed as an extension of the JSP by relaxing its strict assumptions. First, instead of requiring the job process on each machine exactly once, the job can be processed on a subset of machines. Moreover, each operation can be processed on a candidate set of machines. A number of studies have developed integer-programming-based models for finding exact solutions [9, 12, 19, 42, 51, 52]. Second, further relaxation of the assumption that each machine only processes one job at a time and each operation only requires one machine at a time is also necessary. For example, in some healthcare settings, a single clinician may be responsible for a group of patients simultaneously, and a patient may require multiple resources and medical staff at the same time within a single step of medical service. In this context, each machine (clinician) can process more than one job, and each job requires more than one machine. However, to the best of our knowledge, this problem has not been addressed in the literature and is the focus of this research.

To handle modeling uncertainties in the healthcare setting while conducting resource allocation optimization, simulation-based models [20, 23], stochastic programming [26, 34, 48], and distributionally robust optimization models [13, 22, 47] have been developed to improve performance. Granja et al. [20] optimizes patient admission sequence with simulated annealing algorithm and linear programming to minimize waiting time and improve diagnostic imaging workflows. Kim et al. [26] shows that the two-stage stochastic model can lead to significant cost savings compared with the deterministic model for integrated staffing and scheduling for nurse management under demand uncertainty. With time-indexed formulation, Mandelbaum et al. [34] developed an offline appointment sequencing robust optimization approach to reduce 15% - 40% waiting time plus overtime in a clinic’s infusion units. Deng et al. [13] formulates distributionally robust chance constraints for the stochastic surgery planning problem to minimize waiting and operating room (OR) over time under limited data conditions. He et al. [22] proposed a hybrid, robust, stochastic, rank-based formulation of the patient scheduling problem in the Emergency Department and obtained a near-optimal solution. However, they simplify the patient pathways into a queuing network with one waiting pool and only consider the activity event of physicians during the encounter with the patient. Similarly, other recent stochastic scheduling and sequencing literature have not considered the whole path of the patient care and their interactions with different types of staff and resources.

We note that the models mentioned above mainly fall into the time-indexed and rank-based categories, which are intractable in the larger-scale settings that consider all the clinical activities encountered during a patient visit. To address this issue, we propose a new two-stage Stochastic Mixed-Integer Linear Programming (SMILP) formulation without time-indexed or rank-indexed variables for clinical resource allocation and sequencing problems under random activity duration. The proposed SMILP allows for utilizing real-time location data that provide high precision and granularity. We employ the Monte Carlo Optimization (MCO) approach to obtain near-optimal solutions to the SMILP model via its Sample Average Approximation (SAA). Due to its desirable convergence properties, the SAA approach is a popular method for data-driven decision-making problems under a stochastic environment. Our work will apply SAA to tackle the uncertainty challenges in solving complex resource allocation and sequencing problems in the specific context of optimizing patient care pathways. We refer to the references [27, 28, 33, 45] therein for the technical details and discussions on SAA.

## Methods

### Definitions and assumptions

Consider a healthcare setting (e.g., a clinic) where there is a set $\mathcal {J}$ of medical resources (including personnel and equipment), and a set $\mathcal {I}$ of patients that need to be served within service hours [0, *u*]. Given the scheduled appointment time of patients’ clinic visits, our research aims to determine the optimal resource allocation and sequence assignment of the medical activities required to meet patients’ needs within the planning horizon *u*. Each resource in $\mathcal {J}$ belongs to a certain type *g*, where $g\in \mathcal {G}$ and $\mathcal {G}$ is the collection of all resource types, such as specialist, nurse, exam room and equipment. We also define a collection of sets $\mathcal {J}_g$ where $g\in \mathcal {G}$, where each set $\mathcal {J}_g$ contains all the resources that belong to type *g*. For simplicity, let $\mathcal {G} = \{1,2,\cdots , |\mathcal {G}|\}.$

Associated with each scheduled patient, there is a list of activities that need to be completed in a predetermined order, where each activity has a random duration and requires one or more resources. We denote an aggregated list of all patient activities as $\mathcal {A}$. Each activity *a* in $\mathcal {A}$ represents only one particular activity for one patient (not shared by different patients). We denote $\mathcal {A}_0$ as the set of all initial activities for each patient. In addition, we let $\mathcal {A}_1$ represent the set of all activities, excluding the first activities. Therefore, we have $\mathcal {A}= \mathcal {A}_0 + \mathcal {A}_1$. Moreover, we define a function *pre*(*a*) that describes the precedence mapping relationship between activity *a* and its immediate predecessor, where $a\in \mathcal {A}_1$. To link the activities with their resource requirements, we define a collection of sets $\mathcal {A}^{(g)}$, which includes patients’ activities requiring a resource of type *g*, where $g\in \mathcal {G}$.

Next, we define the parameters of the model. For each activity $a\in \mathcal {A}$, we define a non-negative random variable $d_a$ as the service duration of activity *a*, which is independent of the specific resource type serving the activity. To be noted, $d_a$ is a random parameter due to the heterogeneity of patients and the changing dynamics of the healthcare setting. We define scheduled time as $t_a$, where $a\in \mathcal {A}_0$ corresponds to the initial activity for each patient. The time-related parameters, $u,\ d_a,\ t_a$, are integers (in minutes). We define the activity-resource mapping matrix as $\textbf{V}$ with $|\mathcal {A}|$ rows and $|\mathcal {G}|$ columns. The integer element at the *a*th row and *g*th column in matrix $\textbf{V}$, denoted as $V_{a,g}$, represents the number of type *g* resources that are needed for activity *a*. Finally, we denote integer parameters $k_j$ as the capacity of resource *j*, where $j\in \mathcal {J}$.

In the following, we introduce the decision variables for modeling the problem. To represent which resources are assigned to each activity, we define the binary decision variable $x_{j}^a$ that is equal to 1 if resource *j* serves activity *a* and is 0 otherwise, for all pairs (*a*, *j*) where *a* requires the resource of type *j*; mathematically, $\exists g\in \mathcal {G}$, s.t., $j \in \mathcal {J}_g$ and $a \in \mathcal {A}^{(g)}$. For each $a\in \mathcal {A}$, we define a non-negative continuous decision variable $b_{a}$ to represent the beginning time of activity *a*. We define the binary decision variable $s_{1}^{a,a'}$ equals 1 if activity *a* is not assigned before activity $a'$ starts, and is 0 otherwise. We define the binary decision variable $s_{2}^{a,a'}$ that is equal to 1 if activity *a* is assigned before activity $a'$ ends, and is 0 otherwise. For both $s_{1}^{a,a'}$ and $s_{2}^{a,a'}$, we only consider the activity pairs $(a,a')$ that share at least one resource of the same type; mathematically, $\exists g\in \mathcal {G}$, s.t, $a\in \mathcal {A}^{(g)}$ and $a^{\prime }\in \mathcal {A}^{(g)}$. Also, the two activities in the pair $(a, a')$ should not be the same activity. Note that only if $s_{1}^{a,a'}$ and $s_{2}^{a,a'}$ are both equal to 1, it means that activity $a^\prime$ is still ongoing at the time activity *a* starts. Otherwise, if $s_{1}^{a,a'}$ is 0 and $s_{2}^{a,a'}$ is 1, this means that when activity *a* starts, activity $a'$ has not started yet; if $s_{1}^{a,a'}$ is 1 and $s_{2}^{a,a'}$ is 0, this indicates that when activity *a* starts, activity $a'$ has already ended (including those that have just ended). The reason we use decision variables $s^{a,a'}_1$ and $s^{a,a'}_2$ is to determine the activity sequence between each pair of activities sharing the same type of resource, which further helps us to establish capacity constraints for each resource used by activity *a*. Along with resource allocation decision $x_j^a$ and $x_j^{a'}$, we can determine which activity $a'$ is sharing at least one common resource at the time that we start the activity *a*. To do this, we define binary variables $q_{a,a'}^j$ that is equal to 1 if and only if activity $a'$ is in progress at the time activity *a* is assigned and both activities require resource *j*, and is 0 otherwise; mathematically, we have $\exists g\in \mathcal {G}$, s.t, $j \in \mathcal {J}_g$, $a\in \mathcal {A}^{(g)}$ and $a^{\prime }\in \mathcal {A}^{(g)}$. The purpose of $q_{a,a'}^j$ is to subsequently count the total number of ongoing activities sharing the same resource *j* required by the activities so that we can gather information about the serving capacity in use for each resource *j* at the time *a* starts. By constraining the summation of $q_j^{a,a'}$ over $a'$, our model can guarantee that all resources *j* required by *a* have enough remaining capacity to serve this new activity when it starts. Compared to the time-index-based formulation, our formulation simplifies the capacity constraint check as it only needs to be verified at the start of an activity.

Table 1 summarizes all the notations.

**Table 1:** Notations

**Sets**	
$\mathcal {I}$	set of patients to be scheduled,
	$\mathcal {I} = \{0,\cdots , |\mathcal {I}|-1$}
$\mathcal {J}$	set of resources or personnel to be assigned,
	$$\mathcal {J} = \{0,\cdots , |\mathcal {J}|-1\}$$
$\mathcal {G}$	set of the types of resources or personnel
	required, $\mathcal {G} =\{ 0,\cdots , |\mathcal {G}|-1\}$
$\mathcal {J}_g$	sets of resources that belong to type *g*,
	where $g\in \mathcal {G}$
$\mathcal {A}$	set of patients’ activities, $\mathcal {A} =\{ 0,\cdots , |\mathcal {A}|-1\}$
$\mathcal {A}_0$	set of patients’ initial activity
$\mathcal {A}_1$	set of patients’ subsequent activities
$\mathcal {A}^{(g)}$	set of patients’ activities requiring a resource
	of type *g*, where $g\in \mathcal {G}$
**Parameters**	
$t_a$	scheduled time for initial activity *a*,
	where $a\in \mathcal {A}_0$
$d_a$	duration of activity *a*, where $a\in \mathcal {A}$
$V_{a,g}$	the number of type *g* resources required
	by activity *a*, where $g\in \mathcal {G}$ and $a\in \mathcal {A}$
$k_j$	The capacity of resource *j*, where $j\in \mathcal {J}$
**Decision variables**	
$x_{j}^a$	binary assignment variable indicating whether
	resource *j* serves activity *a*, for all pairs (*a*, *j*)
	where *a* requires the resource type of *j*, i.e.,
	$\exists g\in \mathcal {G}$, s.t., $j \in \mathcal {J}_g$ and $a \in \mathcal {A}^{(g)}$.
$b_a$	the actual beginning time of activity $a\in \mathcal {A}$
$s_1^{a,a^{\prime }}$	binary variable indicating whether activity *a* is
	not assigned before activity $a'$, where *a* and $a'$
	share at least one resource of same type $g \in \mathcal {G}$,
	i.e., $\exists g\in \mathcal {G}$, s.t, $a\in \mathcal {A}^{(g)}$ and $a^{\prime }\in \mathcal {A}^{(g)}$
$s_2^{a,a^{\prime }}$	binary variable indicating whether activity *a* is
	assigned before activity $a'$ ends, where *a* and $a'$
	share at least one resource of the same type,
	$g \in \mathcal {G}$ i.e., $\exists g\in \mathcal {G}$, s.t, $a\in \mathcal {A}^{(g)}$ and $a^{\prime }\in \mathcal {A}^{(g)}$
$q_{a,a^{\prime }}^j$	binary variable indicating whether activity $a'$
	is still ongoing when the activity *a* starts and
	both require the same resource *j* at that time,
	i.e., $\exists g\in \mathcal {G}$, s.t, $j \in \mathcal {J}_g$, $a\in \mathcal {A}^{(g)}$ and $a^{\prime }\in \mathcal {A}^{(g)}$

We make the following practical assumptions to complement the model setup:

1. We can obtain the pathway type and its associated activity details for each scheduled patient based on historical data.
2. There are sufficient resources to serve all scheduled patients within the time horizon.
3. Once an activity ends, the patient will wait until the next activity begins.
4. We only consider the segment of patient waiting time after their scheduled appointment time.

Based on the settings and assumptions above, our clinic resource allocation and sequencing model will assign different types of clinicians, staff, and resources to the sequential activities of each patient at specific time points to satisfy their clinic visit requirements while minimizing the expected total waiting time for all patients. Our model will determine the optimized medical personnel assignment, resource allocation, and time sequencing of each activity.

### Optimization models

In this section, we first present the two-stage SMILP formulation in Section 3.2.1. Then, in Section 3.2.2, we present the details of the MCO procedure and the SAA formulation.

#### The two-stage stochastic model

We model the resource allocation and sequence assignment problem as a two-stage SMILP formulation with recourse to minimize the total waiting time along the entire path of the visits for all patients scheduled in the system under uncertainty in the duration of each activity of each patient during their visit. Recourse models arise when the decision-maker must make some (first-stage) decisions (e.g., assign activities to resources at the time when the patient appointment is scheduled) before information relevant to uncertainty (e.g., activity duration on the day of service) is available. In contrast, some (second-stage) decisions (e.g., the start time of each activity) can be delayed until this information is available [4]. It is important to recognize that the timing of the decisions is a property of the problem at hand. For example, in our problem, at the planning stage, we need to assign patient care activities to medical staff and resources and determine the sequence of activities on each resource when scheduling patient appointments while respecting the capacity of the available resources (via first-stage decisions $\varvec{x}, \varvec{s}_1, \varvec{s}_2, \varvec{q}$) before we observe the duration of each activity. These decisions represent the planned schedule and mimic the practice of many healthcare settings employing appointment schedules to manage patient appointments. Due to the uncertainty in the durations of activities, the start time ($\varvec{b}$) may deviate from the scheduled time ($\varvec{t}$). Hence, the activity start time is a second-stage variable.

Our SMILP model incorporates the randomness of activity duration, which is denoted as $\varvec{\xi }$ with the known distribution. We assume that the duration of each activity follows a fully known probability distribution. In reality, there are multiple scenarios of activity duration, with the probability distribution representing all possible scenarios. We denote the set of realizations of duration random variables as $\varvec{\widetilde{d}}$. We estimate the probability distribution of activity duration for each patient from their pathway that is discovered using historical data. Our two-stage model is shown in Formulations () and (). In the first stage, we assign activities to resources and sequence them via decisions $\varvec{x}, \varvec{s}_1, \varvec{s}_2$, and $\varvec{q}$. Again, these are planning decisions we make before observing activity duration and thus are not a function of duration scenarios (i.e., remain the same for each scenario). Then, in the second stage, we determine the start time of each activity (represented by decision $\varvec{b}$) on each resource and compute the waiting time of each activity for each scenario of the activity duration and fixed ($\varvec{x}, \varvec{s}_1, \varvec{s}_2, \varvec{q}$). All inequalities involving the start time decision variables $\varvec{b}$ are placed in the second stage model () since the optimal start time assignment cannot be determined before the specific activity duration realizations. All remaining inequalities without $\varvec{b}$ are included in the first stage model (), which determines the optimal assignment to minimize the expectation of the total waiting time based on the duration distribution $\varvec{\xi }$ without the specific realizations.

Our first stage formulation is as follows.

1a $$\begin{aligned}&v = \min _{\varvec{x},\varvec{s_1},\varvec{s_2},\varvec{q}} f:=\mathbb {E}_{\varvec{\xi }}\left[ Q(\varvec{x},\varvec{s_1},\varvec{s_2},\varvec{q},\xi )\right] \end{aligned}$$

1b $$\begin{aligned} \text {s.t. }&V_{a,g} = \sum _{j\in \mathcal {J}_g} x_j^a, \qquad \qquad \qquad \quad \ \forall a\in \mathcal {A},g\in \mathcal {G}\end{aligned}$$

1c $$\begin{aligned}&q_{a,a^{\prime }}^j \ge s_{1}^{a,a^{\prime }}+s_2^{a,a^{\prime }} + x_j^a + x_j^{a^{\prime }} - 3, \nonumber&\\&\qquad \qquad \ \forall g\in \mathcal {G},\ j\in \mathcal {J}_g,\ a\in \mathcal {A}^{(g)},\ a^{\prime }\in \mathcal {A}^{(g)} \end{aligned}$$

1d $$\begin{aligned}&\sum _{a^{\prime }\in \mathcal {A}^{(g)},a^{\prime } \ne a} q_{a,a^{\prime }}^j \le k_j-1, \nonumber \\&\qquad \qquad \qquad \qquad \quad \forall g\in \mathcal {G},\ j\in \mathcal {J}_g,\ a\in \mathcal {A}^{(g)} \end{aligned}$$

The objective (1a) aims to find optimal first stage decisions ($\varvec{x}, \varvec{s}_1, \varvec{s}_2, \varvec{q}$) that minimize the expected total waiting time. Constraint (1b) is the resource assignment constraint, which guarantees that the resource requirements of each activity will be satisfied. Constraints (1c)–(1d) ensure that activities are sequenced without violating resource capacity constraints. Specifically, constraint (1c) ensures that variables $q_{a,a'}^j$ will equal 1 if and only if all binary variables $s_1^{a,a'}$, $s_2^{a,a'}$, $x_j^{a}$, $x_j^{a'}$ are equal to 1. In other words, constraint (1c) guarantees that $q_{a,a'}^j = 1$ if activity $a'$ is still in progress with resource *j* when activity *a* is scheduled with *j*. Constraint (1d) ensures that the total number of activities being served by resource *j* does not exceed the capacity. These capacity constraints prevent activity *a* from being scheduled to a resource when the resource has already reached its capacity limit.

In the second stage, we compute the beginning time of each activity and the associated waiting time (if any). Recall that we use $\xi$ to represent a realization of a vector of activity duration. For each $\xi$ and feasible ($\varvec{x}, \varvec{s}_2, \varvec{s}_2, \varvec{q}$), our second stage problem is as follows:

2a $$\begin{aligned}&\min _{\varvec{b}}\left\{ \sum _{a\in \mathcal {A}_0}\left( b_a - t_a\right) + \sum _{a\in \mathcal {A}_1}\left( b_a - b_{pre(a)} - \widetilde{d}_{pre(a)}\right) \right\} \nonumber \\ :=&\ Q(\varvec{x},\varvec{s_1},\varvec{s_2},\varvec{q},\xi ) \end{aligned}$$

2b $$\begin{aligned} \text {s.t. }&b_a- t_a \ge 0, \qquad \qquad \qquad \qquad \qquad \quad \forall a \in \mathcal {A}_0 \end{aligned}$$

2c $$\begin{aligned}&b_{a} - b_{pre(a)} - \widetilde{d}_{pre(a)}\ge 0, \qquad \qquad \quad \forall a \in \mathcal {A}_1 \end{aligned}$$

2d $$\begin{aligned}&Ms_{1}^{a,a^{\prime }} \ge b_{a}-b_{a^{\prime }}+1, \nonumber \\&\qquad \qquad \qquad \qquad \forall g\in \mathcal {G},\ a\in \mathcal {A}^{(g)},\ a^{\prime }\in \mathcal {A}^{(g)} \end{aligned}$$

2e $$\begin{aligned}&M(1-s_{1}^{a,a^{\prime }}) \ge b_{a^{\prime }}-b_{a}, \nonumber \\&\qquad \qquad \qquad \qquad \ \forall g\in \mathcal {G},\ a\in \mathcal {A}^{(g)},\ a^{\prime }\in \mathcal {A}^{(g)} \end{aligned}$$

2f $$\begin{aligned}&Ms_2^{a,a^{\prime }} \ge b_{a^{\prime }}-b_{a}+\widetilde{d}_{a^{\prime }}, \nonumber \\&\qquad \qquad \qquad \qquad \ \forall g\in \mathcal {G},\ a\in \mathcal {A}^{(g)},\ a^{\prime }\in \mathcal {A}^{(g)} \end{aligned}$$

2g $$\begin{aligned}&M(1-s_2^{a,a^{\prime }}) \ge b_{a}-b_{a^{\prime }}-\widetilde{d}_{a^{\prime }}+1, \nonumber \\&\qquad \qquad \qquad \qquad \ \forall g\in \mathcal {G},\ a\in \mathcal {A}^{(g)},\ a^{\prime }\in \mathcal {A}^{(g)} \end{aligned}$$

This formulation seeks to minimize the waiting time by assigning the specific starting time (second-stage decision variables) of each activity based on the first-stage assignment and the realized activity duration $\varvec{\widetilde{d}}$. Constraints (2b)–(2c) require that the beginning time of each activity *a* be no earlier than the scheduled start time $t_a$ if *a* is an initial activity, otherwise no earlier than the completion time of its preceding activity (i.e., $b_{pre(a)} + \widetilde{d}_{pre(a)}$). Constraints (2d)–(2g) require that the beginning time of activity *a* obey the first-stage sequencing assignment indicated by $s_1^{a,a'}$ and $s_2^{a,a'}$. Specifically, when $s_1^{a,a'}=1$ (i.e., $a'$ assigned no later than *a*), constraint (2e) reduces to $b_a \ge b_{a'}$, indicating that the second-stage decision for the start time of activity *a* is no earlier than that of activity $a'$, and constraint (2d) is relaxed to $M\ge b_{a} - b_{a^\prime }+ 1$, which is always valid for sufficiently large *M*. In contrast, when $s_1^{a,a'}=0$, constraint (2d) reduced to $b_{a'} \ge b_{a} + 1$, ensuring that activity $a'$ starts later than the start time of activity *a*, and constraint (2e) is relaxed. Similarly, when $s_{2}^{a,a'} = 1$, constraint (2f) is relaxed, while constraint (2g) is reduced to $b_{a}\le b_{a^{\prime }}+\widetilde{d}_{a^{\prime }}-1$, ensuring that the second-stage decision for the start time of activity *a* should be strictly less than the end time of activity $a'$; otherwise, constraint (2g) is relaxed, while constraint (2f) is reduced to $b_{a}\ge b_{a^{\prime }}+\widetilde{d}_{a^{\prime }}$, meaning that activity *a* should start no earlier than the end of activity $a'$.

Our proposed two-stage decision model is a realistic fit for healthcare delivery settings since it is necessary to assign patients to medical staff and resources in advance when the patient is scheduled and before realizing activity duration. Appendix B provides an illustration of the deterministic counterpart of the model.

#### Monte Carlo optimization

Finding the exact optimal solution and objective function value *v* to the two-stage stochastic programming model in Formulations () and () is computationally intractable, since computing $\mathbb {E}_{\varvec{\xi }}\left[ Q(\varvec{x},\varvec{s_1},\varvec{s_2},\varvec{q},\xi )\right]$ requires multidimensional integrals. To obtain a near-optimal solution, we apply MCO with the SAA approach to solve the problem. The key idea of MCO is to replace the duration randomness $\varvec{\xi }$ with the empirical distribution based on *N* i.i.d. sampled scenarios over the original distributions $\varvec{\xi }$. Hence, we can write the SAA transformation of the two-stage model into Formulation (), where we map all previous scenario-related parameters $\widetilde{d}_a$’s and scenario-induced decision variables $b_a$’s into *N* sets of different $d_a^{(n)}$’s and $b_a^{(n)}$’s, where $n = 1, ..., N$. As the weight of each scenario in SAA is equal to 1/*N*, the new objective function becomes the average total waiting time over all the scenarios, shown in Objective (3a). As for the SAA constraints, on the one hand, we can directly reuse the constraints in Formulation () since they do not involve scenario variables; on the other hand, we need to include constraints in Formulation () *N* times. The SAA formulation can now be stated as follows.

3a $$\begin{aligned} v_N :=&\min _{\varvec{x},\varvec{s_1},\varvec{s_2},\varvec{q},\varvec{b}} f_N \nonumber \\ :=&\min _{\varvec{x},\varvec{s_1},\varvec{s_2},\varvec{q},\varvec{b}} \Bigg \{\frac{1}{N}\sum _{n=1}^N \bigg [ \sum _{a\in \mathcal {A}_0}\left( b_a^{(n)} - t_a\right) +\nonumber \\&\qquad \qquad \ \sum _{a\in \mathcal {A}_1}\left( b_a^{(n)} - b^{(n)}_{pre(a)} - d^{(n)}_{pre(a)}\right) \bigg ]\Bigg \} \end{aligned}$$

3b $$\begin{aligned} \text {s.t. }&\text {(1b) -- (1d)} \end{aligned}$$

3c $$\begin{aligned}&\text {(2b) -- (2g)}, \qquad \qquad \qquad \qquad \forall n = 1, ..., N \end{aligned}$$

Note that the sample average $f_N$ in the objective of SAA formulation is an unbiased estimator of the expected value $f:=\mathbb {E}_{\varvec{\xi }}\left[ Q(\varvec{x},\varvec{s_1},\varvec{s_2},\varvec{q},\xi )\right]$ in the objective of the SMILP. By the Law of Large Numbers and [44], $f_N \rightarrow f$ with probability one (w.p.1) as $N \rightarrow \infty$. It follows that we have $v_N \overset{p}{\rightarrow }\ v$ as $N\rightarrow \infty$, i.e., the optimal value of the SAA problem converges to that of the SMILP. However, for a fixed *N*, the SAA formulation reduces to a MILP. Hence, one would expect the computational effort and solution time of solving the SAA formulations to increase as *N* grows. Therefore, we employ the MCO procedure to determine the appropriate sample size *N* to obtain near-optimal solutions that provide a good trade-off between approximation accuracy and computational time.

Algorithm 1 summarizes the steps of the MCO procedure. MCO is able to find an appropriate sample size *N* of duration scenarios which could lead to a near-optimal first-stage decision-making solution within a threshold of the optimal gap. Starting with an initial SAA sample size $N = N_0$, where $N_0$ is far smaller than the pre-defined simulation sample size $N'$, the MCO algorithm first runs *K* replicates of optimization + simulation, in each of which $N+N'$ i.i.d. samples $\varvec{d}^{(n)}\,(n\in [N+N'])$ are drawn from the duration distributions $\varvec{\xi }$; then, the SAA formulation is executed, given the first *N* sampled duration scenarios, which outputs the optimal objective value $v^k_N$ and the first-stage decision solution in this optimization step; next, the SAA formulation is rerun by replacing *N* with $N'$ and changing another set of parameters, that is, the last $N'$ sampled duration scenarios and the fixed first-stage decision parameter gained from the previous optimization step; we then obtain the simulation objective value $v^k_{N'}$. After the *K* replicates, we compute the average optimization-stage objective value $\bar{v}_N$ and the average simulation-stage objective value $\bar{v}_{N'}$. It has been shown that $\bar{v}_N$ and $\bar{v}_{N'}$ are, respectively, the statistical lower and upper bounds of the optimal two-stage model [32].

To evaluate the optimality of the optimization-stage solution under the current sample size *N*, the metric Approximate Optimality Index (AOI) is applied, which is defined by $AOI_N = (|\bar{v}_{N'}-\bar{v}_{N}|)/\bar{v}_{N'}$. If the $AOI_N$ is smaller than the pre-determined termination optimality tolerance $\epsilon$, then we can directly output the results, including the final optimization step *N*, $AOI_N$, the statistical descriptions of lower bound $v^k_{N}$ and upper bound $v^k_{N'}$, and the computational complexity metrics; otherwise, the algorithm will go onto the iteration starting from the scenario generation step until the final $AOI_N$ is smaller than the desired threshold $\epsilon$.

**Fig. 3:**
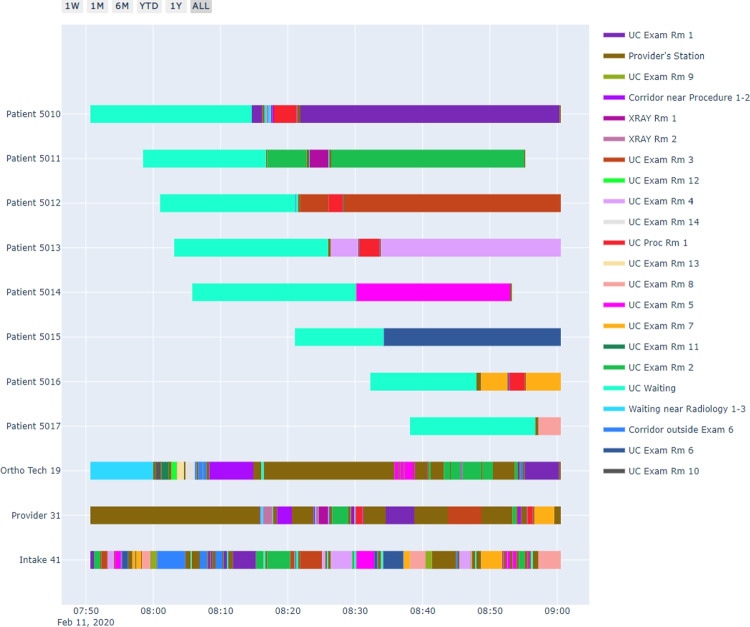
RTLS data visualization example

## Results

In this section, we use the RTLS data to extract the dominant care pathways with a corresponding demand for patient care resources, fit the service time distribution of each care activity for each pathway, and conduct a series of experiments to analyze the computational and operational performance of our SMILP approach. Section 4.1 first describes the RTLS dataset we obtained from our technology partner in Section 4.1.1, then presents the mined pathways in Section 4.1.2, followed by optimization experiment setup in Section 4.1.3. Section 4.2 summarizes the experimental results.

### Data and experiment setup

#### Data description

The data we used in this work was shared by our technology collaborator. We ran experiments using the RTLS data collected from an orthopedics clinic from December 1, 2019, to February 28, 2020. The dataset includes the following four major components:

1. Room information: with attributes of room ID, room name, room location coordinates, and room type;
2. Resource information: with attributes of resource ID, resource type;
3. Patient RTLS data: each row represents a visit activity of a patient in a room, with attributes of patient ID, visit start time, visit end time, move start time (the time of entry into the room), move end time (the time of leaving the room), room name, room ID;
4. Resource RTLS data: each row represents the resource details in a room, with attributes of resource ID, room name, room type, start time, and end time.

#### Discovery of dominant pathways

Patient pathways are chronologically ordered activity se-quences of clinic visits. From the raw RTLS data, we extract the patient-resource interaction events by identifying overlapping periods when patients and resources are in the same location by aggregating patient location data with resource location data. Therefore, each patient’s pathway consists of a sequence of clinical activities, and each of them requires a set of resources in the same room at the same time. When the room changes or the resources that the patient interacts with change, we deem that the patient moves on to the next activity. Notice that even though activity in the pathway is defined based on the co-location interaction with specific types of medical staff, the room is not a scarce resource in this facility setting. Consequently, we do not treat the room as a capacity-limited asset requiring optimization, and it does not directly factor into our experiment’s optimization module. Nevertheless, our SMILP formulation is able to adapt to scenarios where room availability may be restricted, necessitating its consideration as a capacitated resource for optimization.

**Table 2:** Dominant pathways mined from RTLS data

Path ID	Activity list in terms of the interacted resource	Prob	Mean (minutes)	Variance
0	[Intake, Radiology Tech, Provider, Ortho Tech, Discharge]	38.03%	[4.7, 3.48, 4.52, 11.62, 3.43]	[3.25, 4.85, 13.9, 185.52, 2.63]
1	[Intake, Provider, Ortho Tech, Discharge]	24.55%	[4.93, 4.99, 12.47, 3.69]	[4.0, 20.11, 206.96, 2.92]
2	[Intake, Ortho Tech, Discharge]	13.93%	[4.75, 11.82, 3.44]	[4.35, 232.7, 3.36]
3	[Intake, Radiology Tech, Ortho Tech, Discharge]	13.78%	[4.91, 3.58, 11.25, 3.49]	[3.75, 5.79, 224.31, 3.31]
4	[Intake, Radiology Tech, Provider, Discharge]	9.71%	[5.06, 3.52, 6.15, 3.62]	[3.98, 4.95, 30.61, 4.06]

Figure 3 shows an example of RTLS data visualization for Patient 5010, the medical staff Patient 5010 interacted with, and other patients who had interactions with the same set of staff, all within the duration of Patient 5010’s visit. We observe from the plot that different patients do have distinct pathways and medical resource demands; moreover, waiting is a serious problem in this facility.

After data cleaning and preprocessing, we compile all pathways from the raw RTLS data logs of 5644 retrospective clinic visits. The pathways are grouped such that the activity sequences in the same pathway group are exactly the same. The grouping results show that the top 5 types of pathways represent more than 50% of the total visit records. Therefore, we select the top 5 dominant pathway types in our experiments for illustrative purposes and brevity. For the convenience of further pathway sampling, we use the occurrence proportion of each type among these 5 types as the pathway sampling weights.

To determine the characteristics of the duration of each activity for different types of pathways, we use the Python package Scipy to fit the best distributions to the data vectors of activity duration. As shown by May et al. [37], log-normal distributions are the state-of-the-art choice for fitting the actual processing times in clinical settings. Therefore, we fit the log-normal distribution to the duration of each activity type within each pathway type. Our results also confirm that the fitted log-normal distributions are highly consistent with the real data. The dominant pathways and the mean and variance of the fitted distributions are shown in Table 2. During each round of the experiment, we use the dominant pathways to generate a sequence of patients following different pathway patterns. These patterns are sampled using computed proportions. Once the patterns are known, we can identify the mean and variance of duration distribution of each activity for each patient and can further sample different duration scenarios based on the log-normal distribution, which serve as the input to the SAA model.

**Fig. 4:**
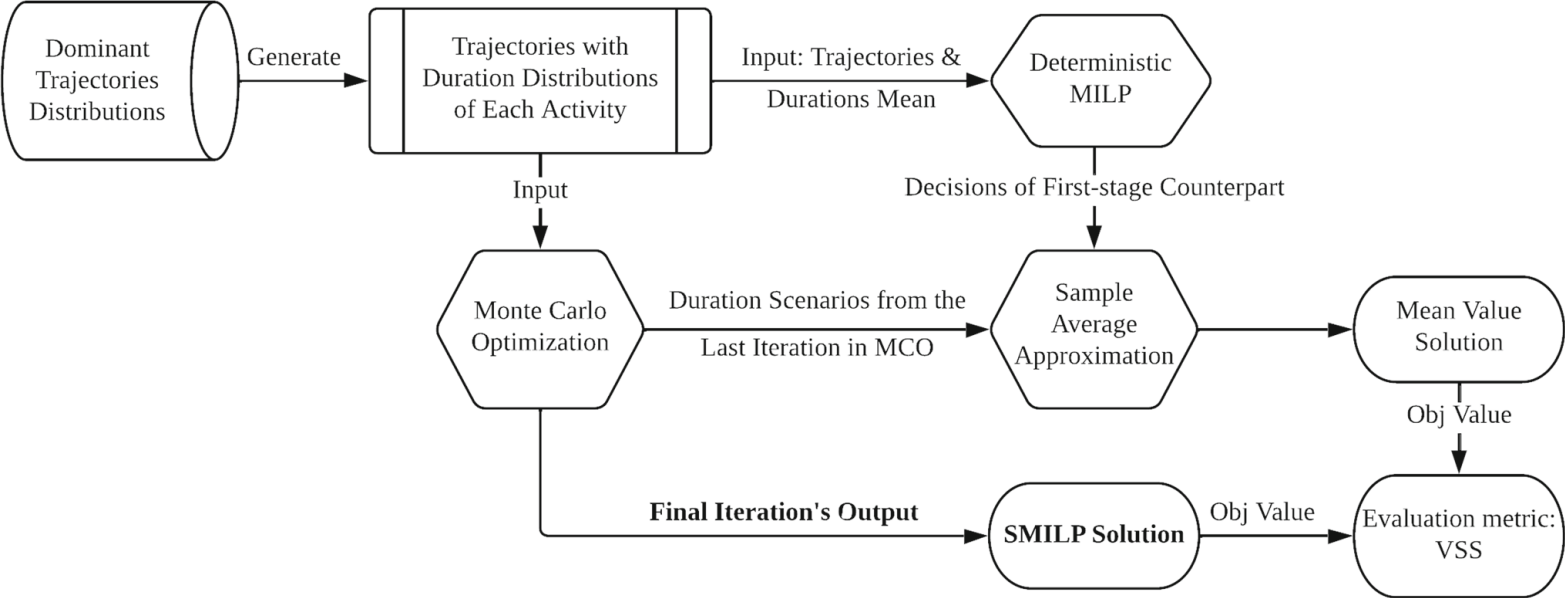
Experiment framework

#### Description of experiment setup

The framework of each trial in each experiment is shown in Fig. 4. Given the dominant patient pathways shown in Table 2, we generate visit instances for patients following these pathways of activity sequences with their known duration distributions weighted by their likelihood of occurrence. This forms the input to the Monte Carlo Optimization approach described in Algorithm 1. Alongside this, we use the mean value of the activity duration in the generated pathways to solve the deterministic counterpart of the proposed SMILP model, where the deterministic solutions of resource allocation and sequencing variables (the counterpart decisions in the first stage of SMILP) will be used for model comparison. The MCO module outputs its simulation scenarios $\varvec{d}_{N'}$ in the last iteration for all the replicates, the average simulation objective value $\bar{v}_{N'}$, and all decision variables’ solution. Note that the model output can also be represented as a resource assignment Gantt chart with the same format as Fig. 6.

For model evaluation, we consider a baseline mean value model whose first-stage solution is derived by the deterministic MILP model under the mean duration setting. Note that this mean value model ignores the randomness in the first stage, which thereby is an appropriate comparison with the SMILP model. For the second stage, we run the SAA model in Formulation () for *K* iterations and obtain *K* objective values. In all *K* replicates, the first-stage variables are fixed with the mean value model solution, while at each iteration, we change the duration scenario with a different scenario from the MCO module’s last iteration. The average of baseline mean value solution objective value is denoted as $\bar{v}_{N'}^{base}$.

Finally, we apply a metric called the value of the stochastic solution (VSS) to measure the value of using SMILP to model the uncertainty in activity duration compared with using a deterministic model, considering only the expected values. VSS is a widely used metric for evaluating stochastic programming models [17]. Specifically, VSS is the difference between the SMILP solution objective value (approximated as $\bar{v}_{N'}$) and the deterministic baseline solution objective value (approximated as $\bar{v}_{N'}^{base}$). Though $\bar{v}_{N'}$ is the approximation of the SMILP solution’s objective value, as we show later, our SAA model provides a small gap for the optimal solution of the SMILP model. Therefore, we use $\bar{v}_{N'}-\bar{v}_{N'}^{base}$ as an estimator of the VSS.

We consider the following combination of key parameters to generate problem instances for our experiments: the number of patients, the arrival sequences of patients with pathway patterns that follow their likelihood of occurrence, patients’ arrival interval, and resource/staff numbers and their capacity. Since we are considering an orthopedics clinic, the scheduling horizon is about 4 hours long. Based on the data summaries, we find that patients arrive at near-equal intervals of around 10 minutes. Hence the average number of hourly arrivals is around 6. Therefore, we follow these interval settings in the design of the experiment by testing two arrival intervals of 10 and 15 minutes each to understand their impact on the total wait time and computational complexity.

On the clinic side, we observe that the main staff types are labeled as Intake, Radiology Technician, Provider, Orthopedics Technician, Discharge, and Others. As Intake and Discharge are not bottleneck activities in the system, we simplify the problem complexity by only evaluating the capacity impact of the Radiology Technician, Provider, and Orthopedics Technician on the clinic’s efficiency, model performance, and computational complexity. For each combination of the parameters, we execute the experiment once based on the framework shown in Fig. 4 to obtain the outputs for different trials. For each trial, we set the initial optimization sample size to $N_0 = 100$ and the simulation optimization sample to $N'=500$.

We executed all the experiments on the Ubuntu 20_04 Devel and Docker operation system with 44 CPUs and a memory size of 120 GB. We employed the CPLEX interface, DOcplex, in Python 3.8 to solve the optimization problem. We used 44 threads to parallelize the computation and set a time limit of 1 hour for solving each optimization problem.

**Fig. 5:**
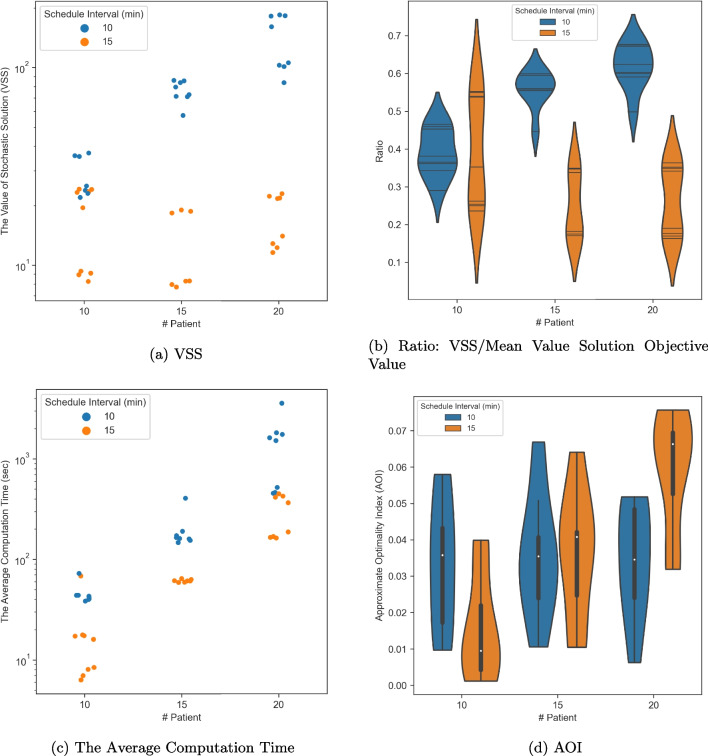
Experiments results: at the iteration of $N = 100$

### Experimental results

Table 3 shows all the MCO procedure’s results based on the settings described in the previous section, where we consider different combinations over multiple parameters. We summarize the results using scatter plots and violin plots for the following metrics:

- The VSS, shown in Fig. 5a;
- The ratio of the VSS over the mean value solution objective value, $\bar{v}_{N'}^{base}$, shown in Fig. 5b;
- The average computation time for the optimization stage, shown in Fig. 5c;
- The Approximate Optimality Index (AOI), shown in Fig. 5d.

In all four subplots, the x-axis delineates the number of patients considered in each experiment, while the color denotes distinct schedule intervals for patient arrivals. In Fig. 5a and c, each point in the scatter plot signifies an experiment setting defined by the number of patients (x-axis), schedule interval (color), patient arrival sequence in terms of pathway patterns, and resource capacity (not shown on these plots); The y-axis represents the corresponding average VSS and average computation time, respectively. Figure 5b showcases violin plots depicting the dual-sided rotated kernel density of the VSS-to-mean-value baseline model objective ratio grouped by patient number (x-axis) and schedule interval (color); Within each violin shape, the vertical position (y-axis) of the horizontal line indicates the actual ratio of an experiment within the group, while the width of the line represents the doubled kernel density at that point. Figure 5d displays a violin plot illustrating the AOI distribution for each experiment set, grouped by the number of patients (x-axis) and the schedule interval (color); This plot, akin to a box plot, incorporates an additional rotated kernel density on each side with trimmed tails beyond extreme values or boxplot range.

For consistent comparative analysis, the results in Fig. 5 are from the first iteration ($N=100$) for each trial. Note that higher VSS and ratio mean better performance of the SMILP model for resource assignment compared to the deterministic solution. The results in Fig. 5a and b indicate that in a more congested setting (shorter schedule interval), our SMILP model can achieve great improvement in the total waiting time compared to the deterministic baseline. The improvement (VSS and the ratio) grows significantly as the number of patients considered in the model (complexity) increases. The total waiting in the system can be reduced by 60% on average with the proposed SMILP model for the most complicated setting (20 patients and an interval of 10 minutes). The most significant reduction in patient waiting time among all the settings is nearly 70%.

From Fig. 5d, we also observe that the SMILP model demonstrates close-to-optimal results for most settings, with the optimality gap smaller than 5%. For the low congestion settings, there is an obvious increase in the AOI as the number of patients increases. One plausible reason is that the low congestion scenarios have more flexibility to assign and sequence the required resources, which results in more computation over multiple resource-pair swappings.

As for the computation time, we conclude from Fig. 5c that the computation time of the optimization stage grows exponentially as the complexity of the model grows (number of patients), noting that the y-axis of Fig. 5c uses the $\log _{10}$-scale. These results are consistent with the literature that the resource allocation problem is NP-hard. Hence, the more congested settings require higher computation time since the model has more constraints. However, we can still obtain near-optimal solutions for the complex model within acceptable time limits that can be implemented in the actual clinic or hospital settings.

## Conclusions, perspectives

This paper focuses on the problem of allocating and sequencing multiple resources so that patients scheduled for clinical care can experience efficient and coordinated care with the shortest overall wait time. We formulate the problem as a two-stage Stochastic Mixed-Integer Linear Programming model. We consider the specific scenario of the resource allocation and sequence assignment that can be conducted by operations staff at the clinic and demonstrate near-optimal solutions for the SMILP problem with different patient arrival sequences generated based on dominant pathways derived from real-time, highly granular, time-stamped location data collected from a single facility. We apply an iterative Monte Carlo Optimization approach for solving the stochastic resource allocation and sequence assignment problem that considers capacitated heterogeneous resources and uncertain service duration for each activity. The objective of the optimization is to minimize the expected total waiting time of all the patients in the clinic during a given planning horizon.

We particularly note that an important innovation in the modeling and solution of this problem is the computationally efficient formulation that uses continuous assignment decision variables without timestamp and position indices, overcoming the significant inefficiencies in traditional formulations with discrete-time-indexed and rank-based (position-indexed) binary assignment decision variables. Within the framework of Monte Carlo Optimization, our Sample Average Approximation formulation realizes fast computation, enabling the solution of the large-scale problem for real-time allocation adjustment in a dynamic clinical environment with complex coordination constraints. We verify the feasibility and superior performance of the proposed model using RTLS data provided by our technology partner. With a detailed design of experiments, the results indicate that our proposed model can significantly reduce waiting time for patients in the clinic by 60%, on average, with acceptable computational resource requirements and time complexity.

The following limitations and extensions provide some directions for future studies.

First, the study insights are limited by the availability of data from a single clinic. Hence, expanding the experiments with additional data from multiple clinics will also allow fitting inter-arrival distributions that consider ‘no-shows’ and the early, late, and on-time arrivals of patients, and their variability, instead of using the deterministic values would be an immediate extension of this work that is more realistic in healthcare settings. The current model can fully support these arrival and ‘no-show’ patterns in varied clinic settings to derive new insights for the multi-resource allocation and sequence assignment problem.

Second, the current pathway discovery approach is limited by data availability to identify the top pathway groups using simple mining methods. With additional data, combining our stochastic model with deeper pathway mining and prediction modeling to construct an end-to-end resource allocation solution is a very promising and needed direction. A more advanced time-series prediction model based on the recurrent neural network or attention network may provide superior accuracy for learning the dominant pathways and predicting the duration for both new and existing clinic patients. The more accurate the pathway information, the better the decision-making performance obtained from the near-optimal solution generated by the MCO approach will be.

Third, limitations imposed by the intractability of the stochastic optimization model may be alleviated by developing an innovative closed-loop model incorporating the further impact of the resource allocation decisions on pathway discovery and prediction. Therefore, the self-feedback mechanism may adaptively predict and optimize [15] to realize a better decision-making allocation for solving the multiple-resource allocation and sequence assignment problem.

## Figures and Tables

**Algorithm 1:**
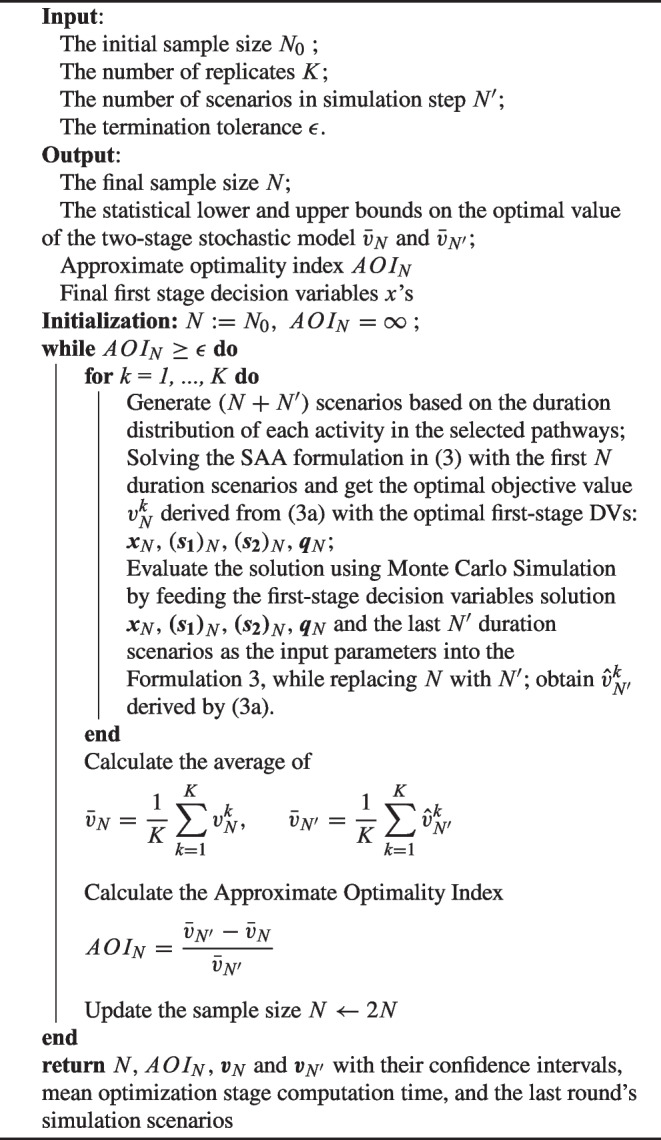
MCO method.

**Fig. 6:**
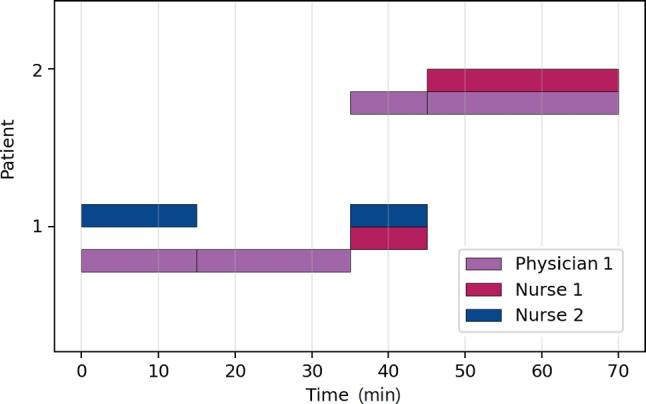
Assignment solution of the deterministic example illustrated in Gantt Chart

## Appendix B: A deterministic illustration

Suppose we want to schedule three resources for two patients to meet their care needs. Assume that the three resources $j_1$ (Physician 1), $j_2$ (Nurse 1), and $j_3$ (Nurse 2) belong to resource group $g_1$ (Physician), group $g_2$ (Nurse), and group $g_2$ (Nurse), respectively; all of them can only serve one patient at a time, which means that their capacity parameters are set as $k_1 = 1,\,k_2=1,\,k_3=1$. Patient 1’s pathway includes 3 activities, denoted as $a_1,\ a_2$, and $a_3$, of duration $d_1 = 15$ minutes, $d_2 = 20$ minutes and $d_3 = 10$ minutes, respectively. Patient 2’s pathway contains 2 activities, denoted as $a_4$ and $a_5$ of duration $d_4 = 10$ minutes and $d_5 = 25$ minutes, respectively. Suppose both patient visits are scheduled at time 0, i.e., $t_1 = t_4 = 0$. Furthermore, we know that $a_1$ requires one physician and one nurse; $a_2$ requires one physician; $a_3$ requires two nurses; $a_4$ requires one physician; $a_5$ requires one physician and one nurse. Therefore, the matrix $\textbf{V}$ can be written as follows

$$ \textbf{V} = \begin{bmatrix} 1 &{} 1\\ 1 &{} 0\\ 0 &{} 2\\ 1 &{} 0\\ 1 &{} 1 \end{bmatrix} $$

The task is to schedule the physician, nurses, and patient to minimize the total waiting time for the patient. The optimal assignment solution is shown in Fig. 6. Specifically, the decision variable (DV) values are as follows: $b_1 =0,\,b_2=15,\,b_3=35,\,b_4=35,\ b_5=45$; all the non-zero binary indicator DVs are as follows: $x_1^1=1,\,x_1^2=1,\,x_1^4=1,\,x_1^5=1,\,x_2^3=1,\, x_2^5=1,\,x_3^1=1,\,x_3^3=1$; $s_1^{4,1} = 1,\,s_1^{4,2} = 1,\,s_1^{5,1} = 1,\,s_1^{5,2}=1,\,s_1^{5,3}=1$; $s_2^{1,4} =1,\,s_2^{2,5}=1,\,s_2^{2,4} =1,\,s_2^{2,5}=1,\,s_2^{3,5}=1$; all *q*’s are 0. Thus the optimal solution indicates that Physician 1 should complete Patient 1’s first and second activities from time 0 to 35 and then switch to serve Patient 2 from time 35 until 70; Nurse 1 should serve Patient 1’s last activity from time 35-45 and serve Patient 2’s last activity from time 45-70; Nurse 2 should serve Patient 1’s first and last activities from time 0-15 and from time 35-45, respectively.

## Appendix C: Experiments and results

The footnotes provide explanations for the columns.^1^.

**Table 3:** Experiments and simulation results

|*I*|	Pathway Pattern	ResourceCapacity	ScheduleInterval(min)	N	Lower	Lower CI	Upper	Upper CI	AOI	Mean obj	MeanTime(sec)	VSS	Ratio
10	[0, 2, 1, 0, 1, 0, 3, 3, 1, 0]	[2, 2, 2]	10	100	41.35	[39.7, 43.01]	42.12	[41.16, 43.08]	1.81%	64.11	42.89	21.99	0.34
10	[0, 2, 1, 0, 1, 0, 3, 3, 1, 0]	[3, 3, 3]	10	100	39.82	[38.67, 40.96]	41.44	[40.6, 42.28]	3.92%	65.33	40.91	23.90	0.37
10	[0, 2, 1, 0, 1, 0, 3, 3, 1, 0]	[4, 4, 4]	10	100	39.35	[38.01, 40.69]	40.76	[39.81, 41.72]	3.47%	65.89	38.34	25.13	0.38
10	[0, 2, 1, 0, 1, 0, 3, 3, 1, 0]	[5, 5, 5]	10	200	39.54	[38.52, 40.56]	41.21	[40.42, 42.01]	4.07%	65.20	123.05	23.99	0.37
10	[0, 2, 1, 0, 1, 0, 3, 3, 1, 0]	[2, 2, 2]	15	100	19.78	[19.08, 20.48]	20.09	[19.75, 20.44]	1.56%	43.42	6.31	23.33	0.54
10	[0, 2, 1, 0, 1, 0, 3, 3, 1, 0]	[3, 3, 3]	15	100	20.12	[19.22, 21.01]	20.03	[19.66, 20.4]	0.42%	43.54	8.04	23.51	0.54
10	[0, 2, 1, 0, 1, 0, 3, 3, 1, 0]	[4, 4, 4]	15	100	19.60	[18.91, 20.3]	19.79	[19.51, 20.08]	0.95%	43.92	6.97	24.12	0.55
10	[0, 2, 1, 0, 1, 0, 3, 3, 1, 0]	[5, 5, 5]	15	100	20.41	[19.58, 21.23]	19.65	[19.3, 19.99]	3.86%	43.88	8.41	24.23	0.55
15	[0, 0, 1, 2, 3, 4, 1, 0, 0, 1, 1, 3, 0, 4, 1]	[2, 2, 2]	10	100	68.34	[66.07, 70.61]	70.99	[69.99, 72.0]	3.74%	128.24	405.50	57.24	0.45
15	[0, 0, 1, 2, 3, 4, 1, 0, 0, 1, 1, 3, 0, 4, 1]	[3, 3, 3]	10	100	55.82	[53.99, 57.65]	57.19	[56.36, 58.02]	2.39%	128.26	171.84	71.07	0.55
15	[0, 0, 1, 2, 3, 4, 1, 0, 0, 1, 1, 3, 0, 4, 1]	[4, 4, 4]	10	100	56.02	[54.32, 57.71]	57.39	[56.77, 58.01]	2.39%	128.75	164.72	71.36	0.55
15	[0, 0, 1, 2, 3, 4, 1, 0, 0, 1, 1, 3, 0, 4, 1]	[5, 5, 5]	10	100	56.76	[55.1, 58.42]	57.37	[56.43, 58.31]	1.06%	130.20	159.41	72.82	0.56
15	[0, 0, 1, 2, 3, 4, 1, 0, 0, 1, 1, 3, 0, 4, 1]	[2, 2, 2]	15	100	34.98	[33.83, 36.13]	35.35	[34.58, 36.13]	1.05%	54.35	60.97	19.00	0.35
15	[0, 0, 1, 2, 3, 4, 1, 0, 0, 1, 1, 3, 0, 4, 1]	[3, 3, 3]	15	100	34.43	[33.33, 35.52]	35.86	[35.22, 36.49]	3.99%	55.36	68.28	19.50	0.35
15	[0, 0, 1, 2, 3, 4, 1, 0, 0, 1, 1, 3, 0, 4, 1]	[4, 4, 4]	15	100	34.45	[33.1, 35.8]	36.00	[35.11, 36.88]	4.29%	54.35	62.94	18.36	0.34
15	[0, 0, 1, 2, 3, 4, 1, 0, 0, 1, 1, 3, 0, 4, 1]	[5, 5, 5]	15	100	34.72	[33.41, 36.03]	35.16	[34.56, 35.76]	1.26%	53.87	64.24	18.71	0.35
20	[4, 0, 4, 4, 1, 0, 2, 2, 1, 1, 0, 0, 0, 0, 3, 4, 2, 0, 0, 1]	[2, 2, 2]	10	100	80.24	[77.14, 83.34]	84.28	[82.94, 85.62]	4.8%	167.98	1753.52	83.70	0.50
20	[4, 0, 4, 4, 1, 0, 2, 2, 1, 1, 0, 0, 0, 0, 3, 4, 2, 0, 0, 1]	[3, 3, 3]	10	100	67.15	[65.13, 69.16]	66.73	[65.74, 67.72]	0.63%	167.68	519.37	100.95	0.60
20	[4, 0, 4, 4, 1, 0, 2, 2, 1, 1, 0, 0, 0, 0, 3, 4, 2, 0, 0, 1]	[4, 4, 4]	10	200	67.16	[66.24, 68.08]	67.54	[66.42, 68.66]	0.56%	169.29	2105.79	101.75	0.60
20	[4, 0, 4, 4, 1, 0, 2, 2, 1, 1, 0, 0, 0, 0, 3, 4, 2, 0, 0, 1]	[5, 5, 5]	10	100	64.92	[63.09, 66.75]	68.31	[67.17, 69.46]	4.97%	170.87	454.71	102.56	0.60
20	[4, 0, 4, 4, 1, 0, 2, 2, 1, 1, 0, 0, 0, 0, 3, 4, 2, 0, 0, 1]	[2, 2, 2]	15	100	39.01	[37.7, 40.32]	40.30	[39.55, 41.05]	3.19%	63.30	168.78	23.01	0.36
20	[4, 0, 4, 4, 1, 0, 2, 2, 1, 1, 0, 0, 0, 0, 3, 4, 2, 0, 0, 1]	[3, 3, 3]	15	200	38.83	[37.92, 39.75]	40.85	[40.06, 41.65]	4.95%	63.39	644.18	22.53	0.36
20	[4, 0, 4, 4, 1, 0, 2, 2, 1, 1, 0, 0, 0, 0, 3, 4, 2, 0, 0, 1]	[4, 4, 4]	15	100	39.35	[38.19, 40.51]	40.76	[40.26, 41.26]	3.46%	62.62	165.38	21.86	0.35
20	[4, 0, 4, 4, 1, 0, 2, 2, 1, 1, 0, 0, 0, 0, 3, 4, 2, 0, 0, 1]	[5, 5, 5]	15	200	39.37	[38.25, 40.48]	40.95	[40.31, 41.6]	3.88%	63.59	628.26	22.63	0.36
10	[0, 0, 0, 2, 2, 0, 0, 3, 3, 2]	[2, 2, 2]	10	200	54.76	[53.76, 55.76]	56.49	[55.19, 57.78]	3.06%	79.53	334.30	23.05	0.29
10	[0, 0, 0, 2, 2, 0, 0, 3, 3, 2]	[3, 3, 3]	10	100	42.48	[41.24, 43.73]	42.08	[40.91, 43.24]	0.97%	77.89	43.80	35.81	0.46
10	[0, 0, 0, 2, 2, 0, 0, 3, 3, 2]	[4, 4, 4]	10	100	40.82	[39.55, 42.09]	42.38	[41.55, 43.21]	3.69%	79.32	43.76	36.94	0.47
10	[0, 0, 0, 2, 2, 0, 0, 3, 3, 2]	[5, 5, 5]	10	100	42.23	[40.89, 43.57]	42.86	[41.81, 43.9]	1.46%	78.34	40.23	35.49	0.45
10	[0, 0, 0, 2, 2, 0, 0, 3, 3, 2]	[2, 2, 2]	15	100	26.15	[24.8, 27.5]	26.18	[25.71, 26.66]	0.12%	35.49	17.24	9.31	0.26
10	[0, 0, 0, 2, 2, 0, 0, 3, 3, 2]	[3, 3, 3]	15	100	26.87	[25.69, 28.04]	26.78	[26.26, 27.29]	0.34%	35.04	17.42	8.26	0.24
10	[0, 0, 0, 2, 2, 0, 0, 3, 3, 2]	[4, 4, 4]	15	100	26.17	[25.15, 27.19]	26.76	[25.91, 27.61]	2.2%	35.69	17.75	8.93	0.25
10	[0, 0, 0, 2, 2, 0, 0, 3, 3, 2]	[5, 5, 5]	15	100	26.51	[25.45, 27.57]	26.75	[26.1, 27.39]	0.87%	35.85	16.02	9.10	0.25
15	[0, 1, 1, 0, 0, 0, 4, 2, 2, 4, 1, 0, 0, 3, 2]	[2, 2, 2]	10	200	60.48	[59.44, 61.52]	60.30	[59.29, 61.31]	0.29%	142.33	1081.64	82.03	0.58
15	[0, 1, 1, 0, 0, 0, 4, 2, 2, 4, 1, 0, 0, 3, 2]	[3, 3, 3]	10	100	55.09	[53.89, 56.3]	57.14	[56.18, 58.09]	3.58%	142.61	161.08	85.47	0.60
15	[0, 1, 1, 0, 0, 0, 4, 2, 2, 4, 1, 0, 0, 3, 2]	[4, 4, 4]	10	200	56.51	[55.3, 57.72]	57.17	[56.07, 58.26]	1.14%	143.39	798.97	86.22	0.60
15	[0, 1, 1, 0, 0, 0, 4, 2, 2, 4, 1, 0, 0, 3, 2]	[5, 5, 5]	10	100	54.98	[53.95, 56.01]	56.98	[56.07, 57.89]	3.51%	140.72	146.77	83.74	0.60
15	[0, 1, 1, 0, 0, 0, 4, 2, 2, 4, 1, 0, 0, 3, 2]	[2, 2, 2]	15	200	37.14	[36.29, 38.0]	37.24	[36.66, 37.82]	0.25%	45.73	228.65	8.49	0.19
15	[0, 1, 1, 0, 0, 0, 4, 2, 2, 4, 1, 0, 0, 3, 2]	[3, 3, 3]	15	100	36.02	[34.68, 37.37]	37.58	[36.64, 38.52]	4.14%	45.32	60.83	7.74	0.17
15	[0, 1, 1, 0, 0, 0, 4, 2, 2, 4, 1, 0, 0, 3, 2]	[4, 4, 4]	15	100	35.73	[34.34, 37.12]	37.25	[36.51, 38.0]	4.08%	45.54	59.11	8.29	0.18
15	[0, 1, 1, 0, 0, 0, 4, 2, 2, 4, 1, 0, 0, 3, 2]	[5, 5, 5]	15	100	36.07	[35.04, 37.11]	37.45	[36.67, 38.22]	3.67%	45.42	61.24	7.97	0.18
20	[3, 0, 1, 1, 1, 3, 0, 3, 2, 0, 2, 1, 3, 0, 0, 0, 0, 0, 0, 0]	[2, 2, 2]	10	100	108.45	[104.54, 112.36]	111.22	[109.75, 112.69]	2.49%	271.87	3586.03	160.65	0.59
20	[3, 0, 1, 1, 1, 3, 0, 3, 2, 0, 2, 1, 3, 0, 0, 0, 0, 0, 0, 0]	[3, 3, 3]	10	100	86.17	[83.84, 88.5]	88.05	[86.8, 89.3]	2.14%	272.56	1820.09	184.51	0.68
20	[3, 0, 1, 1, 1, 3, 0, 3, 2, 0, 2, 1, 3, 0, 0, 0, 0, 0, 0, 0]	[4, 4, 4]	10	100	85.41	[83.47, 87.36]	88.66	[87.72, 89.59]	3.66%	270.46	1521.32	181.81	0.67
20	[3, 0, 1, 1, 1, 3, 0, 3, 2, 0, 2, 1, 3, 0, 0, 0, 0, 0, 0, 0]	[5, 5, 5]	10	100	85.92	[83.31, 88.53]	88.82	[87.52, 90.11]	3.26%	271.49	1623.27	182.67	0.67
20	[3, 0, 1, 1, 1, 3, 0, 3, 2, 0, 2, 1, 3, 0, 0, 0, 0, 0, 0, 0]	[2, 2, 2]	15	200	57.12	[56.09, 58.16]	58.87	[58.23, 59.52]	2.97%	71.16	1518.51	12.29	0.17
20	[3, 0, 1, 1, 1, 3, 0, 3, 2, 0, 2, 1, 3, 0, 0, 0, 0, 0, 0, 0]	[3, 3, 3]	15	400	66.71	[55.56, 77.86]	68.20	[56.8, 79.61]	2.18%	72.72	3607.43	4.51	0.06
20	[3, 0, 1, 1, 1, 3, 0, 3, 2, 0, 2, 1, 3, 0, 0, 0, 0, 0, 0, 0]	[4, 4, 4]	15	200	57.57	[56.74, 58.39]	60.14	[59.46, 60.81]	4.27%	73.80	1752.78	13.66	0.19
20	[3, 0, 1, 1, 1, 3, 0, 3, 2, 0, 2, 1, 3, 0, 0, 0, 0, 0, 0, 0]	[5, 5, 5]	15	200	57.93	[56.67, 59.2]	59.06	[58.28, 59.83]	1.9%	72.37	1764.75	13.32	0.18

## References

[1] Asamoah DA, Sharda R, Rude HN, Doran D (2018) Rfid-based information visibility for hospital operations: exploring its positive effects using discrete event simulation. Health Care Manag Sci 21:305–316 27734237 10.1007/s10729-016-9386-y

[2] Christopher BJ, Feng TK, Watson J-P (2011) Combining constraint programming and local search for job-shop scheduling. INFORMS J Comput 23(1):1–14

[3] Berg B, Longley G, Dunitz J (2019) Improving clinic operational efficiency and utilization with rtls. J Med Syst 43(3):56 30701407 10.1007/s10916-019-1174-z

[4] Birge JR, Louveaux F (2011) Introduction to stochastic programming. Springer Science & Business Media

[5] Boginski V, Mun IK, Wu Y, Mason KP, Zhang C (2007) Simulation and analysis of hospital operations and resource utilization using rfid data. In: 2007 IEEE international conference on RFID, IEEE, pp 199–204

[6] Bohmer R, Pisano G, Sadun R, Tsai T (2020) How hospitals can manage supply shortages as demand surges. Harvard Business Rev 3

[7] Kamel Boulos MN, Berry G (2012) Real-time locating systems (rtls) in healthcare: a condensed primer. Int J Health Geographics 11(1):1–8 10.1186/1476-072X-11-25 PMC3408320 22741760

[8] Bowman EH (1959) The schedule-sequencing problem. Oper Res 7(5):621–624

[9] Brucker P, Schlie R (1990) Job-shop scheduling with multi-purpose machines. Computing 45(4):369–375

[10] Carrasco VN, Jackson SS (2010) Real time location systems and asset tracking: new horizons for hospitals. Biomed Instrum Technol 44(4):318–323 20715958 10.2345/0899-8205-44.4.318

[11] Cayirli T, Veral E (2003) Outpatient scheduling in health care: a review of literature. Prod Oper Manag 12(4):519–549

[12] Imran AC, Abid AK (2016) A research survey: review of flexible job shop scheduling techniques. Int Trans Oper Res 23(3):551–591

[13] Deng Y, Shen S, Denton B (2019) Chance-constrained surgery planning under conditions of limited and ambiguous data. INFORMS J Comput 31(3):559–575

[14] Ding B, Chen L, Chen D, Yuan H (2008) Application of rtls in warehouse management based on rfid and wi-fi. In: 2008 4th International conference on wireless communications, networking and mobile computing, IEEE, pages 1–5

[15] Elmachtoub AN, Grigas P (2021) Smart “predict, then optimize”. Management Science

[16] Emanuel EJ, Persad G, Upshur R, Thome B, Parker M, Glickman A, Zhang C, Boyle C, Smith M, Phillips JP (2020) Fair allocation of scarce medical resources in the time of Covid-19. N Engl J Med 382(21):2049–2055 32202722 10.1056/NEJMsb2005114

[17] Escudero LF, Garín A, Merino M, Pérez G (2007) The value of the stochastic solution in multistage problems. Top 15(1):48–64

[18] Garey MR, Johnson DS, Sethi R (1976) The complexity of flowshop and jobshop scheduling. Math Oper Res 1(2):117–129

[19] Gartner D, Padman R (2020) Flexible hospital-wide elective patient scheduling. J Oper Res Soc 71(6):878–892

[20] Granja C, Almada-Lobo B, Janela F, Seabra J, Mendes A (2014) An optimization based on simulation approach to the patient admission scheduling problem using a linear programing algorithm. J Biomed Inform 52:427–437 25194680 10.1016/j.jbi.2014.08.007

[21] Gupta D, Denton B (2008) Appointment scheduling in health care: challenges and opportunities. IIE Trans 40(9):800–819

[22] He S, Sim M, Zhang M (2019) Data-driven patient scheduling in emergency departments: a hybrid robust-stochastic approach. Manage Sci 65(9):4123–4140

[23] Huang W-T, Chen P-S, Liu JJ, Chen Y-R, Chen Y-H (2018) Dynamic configuration scheduling problem for stochastic medical resources. J Biomed Inform 80:96–105 29548712 10.1016/j.jbi.2018.03.005

[24] Kato-Lin Y-C, Padman R (2019) RFID technology-enabled Markov reward process for sequencing care coordination in ambulatory care: a case study. Int J Inf Manage 48:12–21

[25] Kim J, Ok C, Kumara SRT, Yee S-T (2007) Multiagent-based dynamic deployment planning in rtls-enabled automotive shipment yard. In: Conference of the Canadian society for computational studies of intelligence, Springer, pp 38–49

[26] Kim K, Mehrotra S (2015) A two-stage stochastic integer programming approach to integrated staffing and scheduling with application to nurse management. Oper Res 63(6):1431–1451

[27] Kim S, Pasupathy R, Henderson SG (2015) A guide to sample average approximation. Handbook of Simulation Optimization, pp 207–243

[28] Kleywegt AJ, Shapiro A, Mello TH-de, (2002) The sample average approximation method for stochastic discrete optimization. SIAM J Optim 12(2):479–502

[29] Kong Q, Lee C-Y, Teo C-P, Zheng Z (2016) Appointment sequencing: Why the smallest-variance-first rule may not be optimal. Eur J Oper Res 255(3):809–821

[30] Wen-Yang K, Christopher BJ (2016) Mixed integer programming models for job shop scheduling: a computational analysis. Comput Oper Res 73:165–173

[31] Lin Y-C, Padman R (2013) Process visibility analysis in ambulatory care: a simulation study with rfid data. In: MEDINFO 2013, Stud Health Technol Inform, IOS Press, pp 768–772 23920661

[32] Linderoth J, Shapiro A, Wright S (2006) The empirical behavior of sampling methods for stochastic programming. Ann Oper Res 142(1):215–241

[33] Mak W-K, Morton DP, Kevin WR (1999) Monte carlo bounding techniques for determining solution quality in stochastic programs. Oper Res Lett 24(1–2):47–56

[34] Mandelbaum A, Momčilović P, Trichakis N, Kadish S, Leib R, Bunnell CA (2020) Data-driven appointment-scheduling under uncertainty: the case of an infusion unit in a cancer center. Manag Sci 66(1):243–270

[35] Mangiarotti S, Peyre M, Zhang Y, Huc M, Roger F, Kerr Y (2020) Chaos theory applied to the outbreak of covid-19: an ancillary approach to decision making in pandemic context. Epidem Inf 148 10.1017/S0950268820000990 PMC7231667 32381148

[36] Manne AS (1960) On the job-shop scheduling problem. Operations Research 8(2):219–223

[37] May JH, Spangler WE, Strum DP, Vargas LG (2011) The surgical scheduling problem: current research and future opportunities. Prod Oper Manag 20(3):392–405

[38] Meskó B, Hetényi G, Győrffy Z (2018) Will artificial intelligence solve the human resource crisis in healthcare? BMC Health Serv Res 18(1):1–4 30001717 10.1186/s12913-018-3359-4 PMC6044098

[39] Miller MJ, Ferrin DM, Flynn T, Ashby M, White KP, Mauer MG (2006) Using rfid technologies to capture simulation data in a hospital emergency department. In: Proceedings of the 2006 winter simulation conference, IEEE, pp 1365–1371

[40] Newman-Casey PA, Musser J, Niziol LM, Shedden K, Burke D, Cohn A (2020) Designing and validating a low-cost real time locating system to continuously assess patient wait times. J Biomed Inform 106:103428 32339749 10.1016/j.jbi.2020.103428 PMC7324007

[41] Othman SB, Zgaya H, Hammadi S, Quilliot A, Martinot A, Renard J-M (2016) Agents endowed with uncertainty management behaviors to solve a multiskill healthcare task scheduling. J Biomed Inform 64:25–43 27544412 10.1016/j.jbi.2016.08.011

[42] Özgüven C, Özbakır L, Yavuz Y (2010) Mathematical models for job-shop scheduling problems with routing and process plan flexibility. Appl Math Model 34(6):1539–1548

[43] Salzarulo Peter A, Mahar Stephen, Modi Sachin (2016) Beyond patient classification: using individual patient characteristics in appointment scheduling. Prod Oper Manag 25(6):1056–1072

[44] Shapiro A (2003) Monte carlo sampling approach to stochastic programming. In: ESAIM: Proceedings, EDP Sciences, vol 13, pp 65–73

[45] Shapiro A, Dentcheva D, Ruszczynski A (2021) Lectures on stochastic programming: modeling and theory. SIAM

[46] Shehadeh KS, Cohn AEM, Jiang R (2021) Using stochastic programming to solve an outpatient appointment scheduling problem with random service and arrival times. Naval Research Logistics (NRL) 68(1):89–111

[47] Shehadeh KS, Padman R (2021) A distributionally robust optimization approach for stochastic elective surgery scheduling with limited intensive care unit capacity. European J Oper Res 290(3):901–913

[48] Shehadeh KS, Padman R (2022) Stochastic optimization approaches for elective surgery scheduling with downstream capacity constraints: models, challenges, and opportunities. Comput Oper Res 137:105523

[49] Singh R, Mindel V, Mathiassen L (2017) It-enabled revenue cycle transformation in resource-constrained hospitals: a collaborative digital options inquiry. J Manag Inf Syst 34(3):695–726

[50] Srinivas S, Ravi RA (2018) Optimizing outpatient appointment system using machine learning algorithms and scheduling rules: a prescriptive analytics framework. Expert Syst Appl 102:245–261

[51] Thomalla CS (2001) Job shop scheduling with alternative process plans. Int J Prod Econ 74(1–3):125–134

[52] Torabi SA, Karimi B, Fatemi GSMT (2005) The common cycle economic lot scheduling in flexible job shops: the finite horizon case. Int J Prod Econ 97(1):52–65

[53] Villa S, Barbieri M, Lega F (2009) Restructuring patient flow logistics around patient care needs: implications and practicalities from three critical cases. Health Care Manag Sci 12(2):155–165 19469455 10.1007/s10729-008-9091-6

[54] Wagner HM (1959) An integer linear-programming model for machine scheduling. Naval Res Log Quart 6(2):131–140

[55] Welch JD, Bailey NTJ (1952) Appointment systems in hospital outpatient departments. The Lancet 259(6718):1105–1108 10.1016/s0140-6736(52)90763-0 14928595

[56] Yao W, Chu C-H, Li Z (2012) The adoption and implementation of rfid technologies in healthcare: a literature review. J Med Syst 36(6):3507–3525 22009254 10.1007/s10916-011-9789-8

